# Stem Cell Injection for Complex Refractory Perianal Fistulas in Crohn's Disease: A Single Center Initial Experience

**DOI:** 10.3389/fsurg.2022.834870

**Published:** 2022-02-07

**Authors:** Francesco Colombo, Francesco Cammarata, Caterina Baldi, Francesco Rizzetto, Andrea Bondurri, Stefania Carmagnola, Daniele Gridavilla, Giovanni Maconi, Sandro Ardizzone, Piergiorgio Danelli

**Affiliations:** ^1^Department of Surgery, Luigi Sacco University Hospital, University of Milan, Milan, Italy; ^2^Department of Radiology, ASST Grande Ospedale Metropolitano Niguarda, Milan, Italy; ^3^Department of Gastroenterology, Luigi Sacco University Hospital, University of Milan, Milan, Italy; ^4^Department of Biomedical and Clinical Sciences Luigi Sacco, University of Milan, Milan, Italy

**Keywords:** Crohn's disease, stem cells, perianal abscess and fistula, mesenchymal, postoperative outcomes, inflammatory bowel disease

## Abstract

From 30 to 70% of patients with Crohn's disease (CD) may develop perianal fistulas during their lifetime. The medical and surgical management of this complication is challenging, and its treatment still gives unsatisfactory results. However, recent studies on adipose-derived mesenchymal stem cells have proven their anti-inflammatory and immuno-modulatory potential, representing a new promising tool in the treatment of such stubborn disease. We report our initial experience with three patients who had recurrent perianal CD treated with local infiltration of stem cell darvadstrocel (Alofisel). All the patients had a long history of perianal disease refractory to multiple medical and surgical treatments. The preoperative workup included transperineal ultrasound (TP-US), pelvic MRI, and colonoscopy that ruled out active proctitis in all the patients. The post-treatment follow-up included clinical assessment at 1, 3, and 6 months with repeated MRI and TP-US at 6 months. At 6 months, 2 patients had a clinical response despite radiological persistence of fistula tracts, while one patient presented perianal fistula recurrence complicated by perianal abscess. Although our experience is limited to 3 patients and a short follow-up, our results confirm that darvadstrocel injection is a safe procedure, with a good clinical response in most of the patients, but that it apparently had no effect on the anatomical modification of the fistula tracts. Long-term results, with a rigorous assessment of anatomical lesions, are still needed to support the promising data of the literature.

## Introduction

The perianal fistulizing disease is a striving condition that is quite common in patients with Crohn's disease (CD) during their lifetime. It can sometimes make the therapeutic management of a patient very difficult, requiring multiple surgical procedures but with frequently disappointing results in terms of recurrence and quality of life. Diagnosis and management of perianal CD require an expert, multidisciplinary approach, and even in tertiary inflammatory bowel disease (IBD) centers its treatment remains a challenge, with around 40% of patients experiencing refractory disease.

Usually, at the beginning, a medical approach is attempted with antibiotics, immunomodulators, or biologics, but frequently the symptoms are little controlled with the persistence of pain, discharge, and recurrent abscesses. Surgical intervention remains a cornerstone of management of perianal fistulizing disease with different techniques and approaches proposed in the literature. After adequate medical control of luminal disease, the established goal is to heal the fistula by preservation of fecal continence.

The surgical procedures vary from seton placement, fistulotomy, fistulectomy, endorectal advanced flap formation to fecal diversion or proctectomy. Limited data in the literature are also available on ligation of intersphincteric fistula tract (LIFT) and video-assisted anal fistula treatment of CD (1).

Stem cell therapy is the new kid on the block to be added to the armamentarium of treatments used in perianal CD. It has been demonstrated that immuno-modulatory properties are independent from the site of origin and liposuction remains one of the most convenient ways to harvest the highest possible number of cells. Recent studies on adipose-derived mesenchymal stem cells have proven their anti-inflammatory and immuno-modulatory potential, representing a new promising tool in the treatment of such challenging disease (2, 3). Darvadstrocel is a suspension of expanded adipose stem cells, and is designed to be administered through local injection in the fistula region. They have been studied as direct injection into fistula tracts, incorporated into fibrin glue, or attached to a fistula plug. We report our initial experience with three patients who had recurrent perianal Crohn's disease subjected to local infiltration of stem cell darvadstrocel (Alofisel).

## Selection of Cases

In the period October 2020-February 2021 at Luigi Sacco University Hospital of Milan, a short series of CD patients complicated with perianal CD was selected for treatment with mesenchymal stem cell injection. In particular, the patients were selected among those with a long history and refractory perianal CD, with failure to more than 2 lines of medical therapies (azathioprine, infliximab, adalimumab, etc.) and more than 2 surgical treatments for their perianal disease. We excluded patients with anal strictures and recto-vaginal or anovulvar fistulas, and all patients with active luminal CD of the rectum/anal canal.

## Surgical Procedure

**Figure 1:**
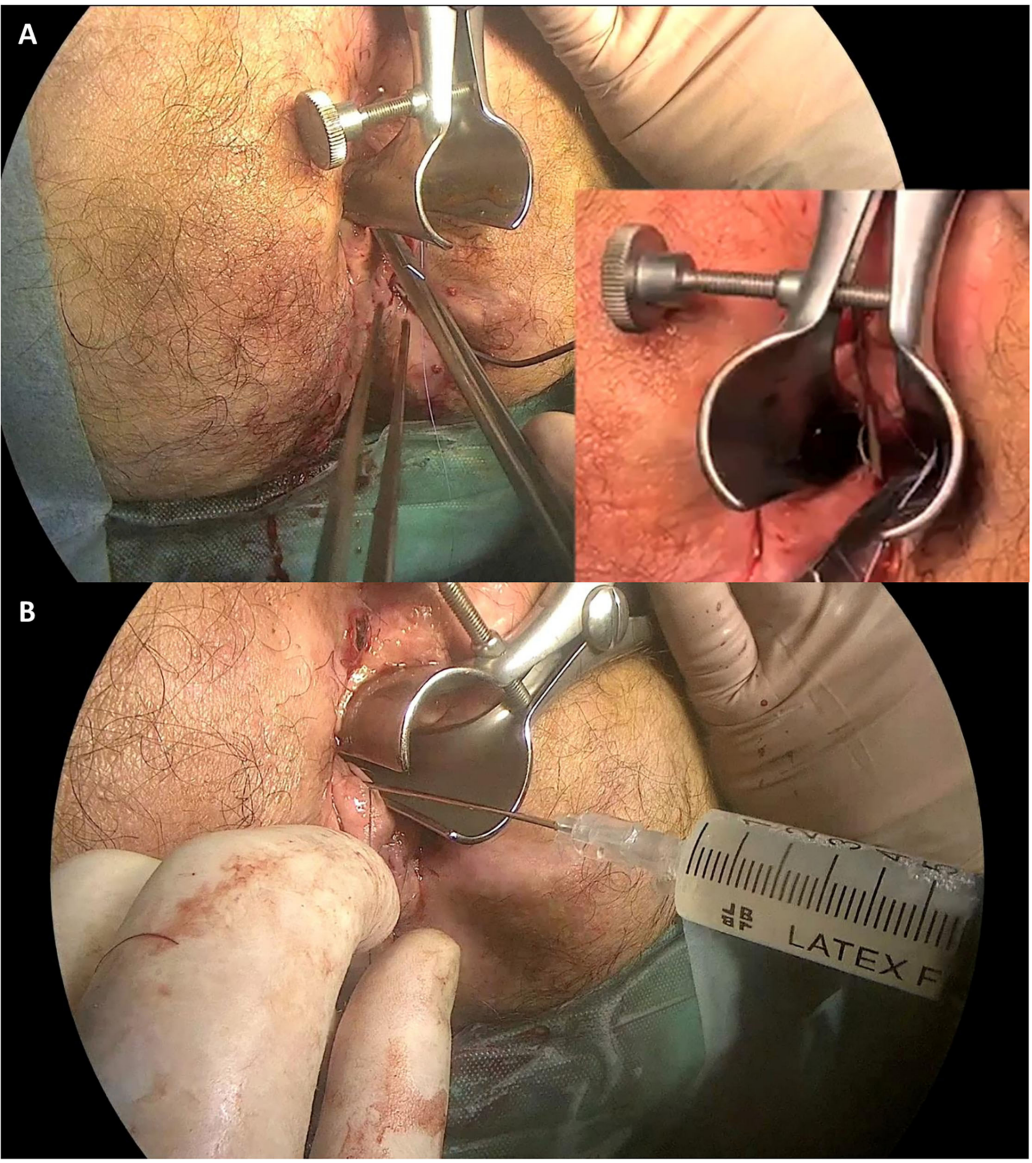
Surgical procedure. Panel **(A)** shows the fistula's internal orifice closure with a 2/0 absorbable stitch. Panel **(B)** shows the stem cell (Darvadstrocel) injection in the tissue around the internal orifice and through the external orifice into the fistula walls.

After providing written informed consent, an examination under general anesthesia (EUA) of the perineal region was performed on each patient. The first step was proper conditioning of all the fistula tracts by identification and mechanical debridement (curettage) of the internal orifice. Especially in the presence of a seton previously positioned, after removing it, extensive de-epithelization was performed with small probes. Then, the tracts were cleaned with a saline solution to remove devitalized tissue debris. The second step was to proceed with the closure of internal fistula opening(s) with a 2/0 absorbable suture. The stitch should include full-thickness bites to seal the orifice (Figure 1A). Finally, we performed the injection of 120 millions of stem cells (darvadstrocel) with a long fine needle (22G). We used a needle for spinal anesthesia measuring around 90 mm in length. Usually, half dose was injected in tissues around the internal orifice while the other half dose was administered through the external orifice into fistula walls throughout all its length, creating several microblebs. Care was taken not to inject the cells neither within the fistula lumen nor too far (> 2–3 mm) from the fistula walls (Figure 1B). The surgical procedure is completed with soft massage of the implant area.

Our step-by-step procedure is similar to a previously described surgical technique in the literature (4).

All the procedures were performed by a single expert surgeon skilled on different IBD procedures. Before the first operation, the surgeon received brief training for stem cell injection.

## Workup and Follow-Up Procedures

We performed on each patient a preoperative MRI and TP-US to characterize the perianal fistulas and their different components, and to identify branches, extensions, and fluid collections of the fistulas, as previously reported (3). A preoperative colonoscopy was also carried out to rule out any sign of proctitis or active luminal disease of the anal canal.

Postoperatively, we assessed all the patients with clinical examination at 1, 3, and 6 months in outpatient clinic. During the assessment, we performed an accurate evaluation of all external openings evaluating spontaneous drainage or drainage after gentle compression as previously described by Present et al. (5). At 6 months, we also repeated the MRI, TP-US, and colonoscopy.

### Case 1

**Figure 2:**
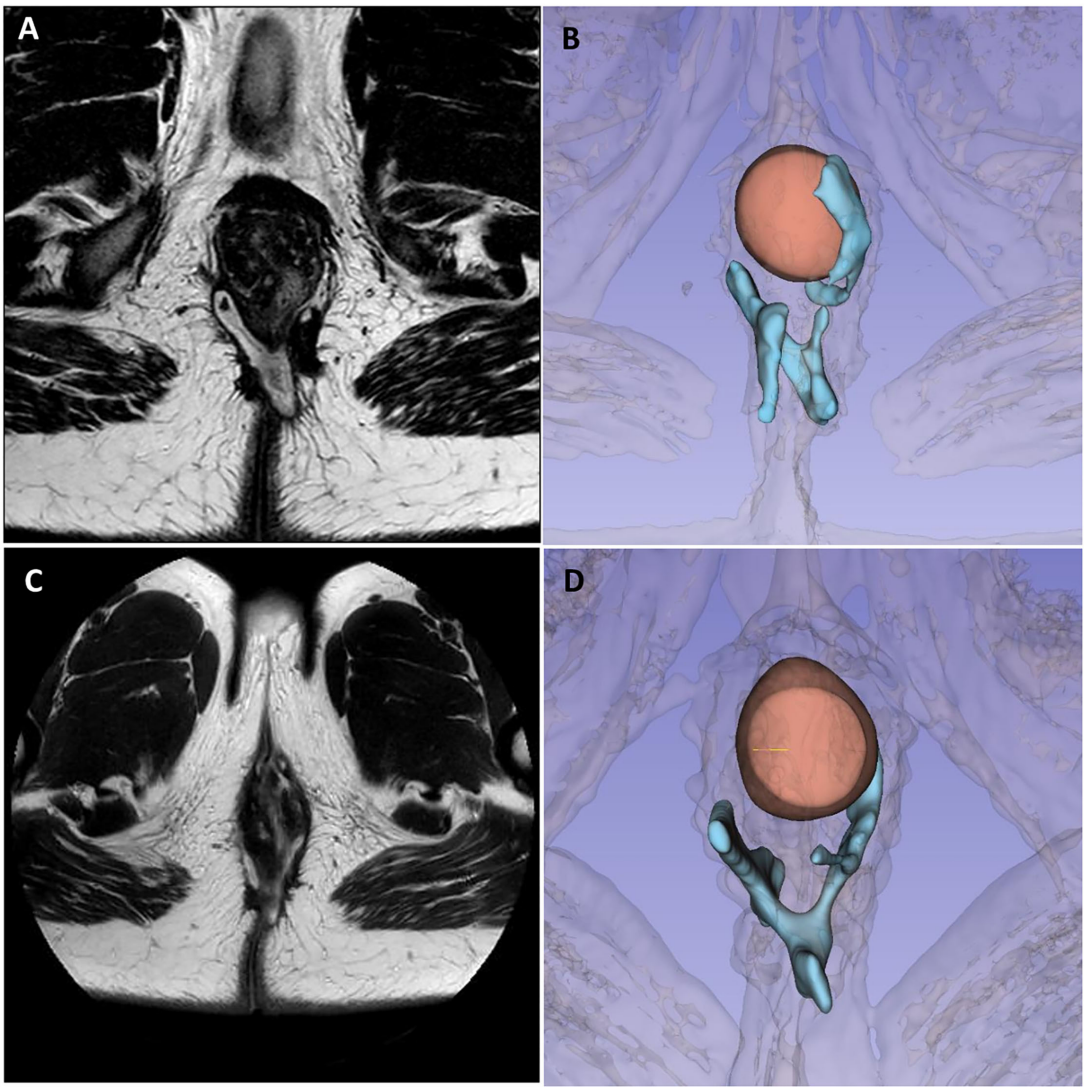
Case 1 imaging pre- and post-surgery. Panels **(A, B)** show preoperative pelvic magnetic resonance imaging and 3D reconstruction highlighting a complex branched perianal fistulising disease with trans-sphincteric fistulas joining in the posterior plane with two external orifices on the right and one on the left, with a 2-cm abscess in the left ischioanal fat. Panels **(C, D)** show 6-month MRI follow-up, with the persistence of fistula tracts, less signs of inflammation, less thickening, contrast enhancement, and no fluid collections.

The first case is about a 26-year-old man with colonic and perianal Crohn's disease [A2L2B1p according to Montreal Classification (6)]. Since adolescent, he has been undergoing multiple perianal abscess drainages, fistulectomies, and seton placements. He was previously treated with adalimumab and then vedolizumab for almost 1 year along with multiple repeated cycles of antibiotic therapy. A preoperative MRI (Figures 2A,B) and TP-US showed a complex perianal disease with branched fistulas: on the right, a trans-sphincteric fistula with two external orifices (h7 and h8) and an internal orifice in the median posterior wall of the rectum; and on the left, a branched trans-sphincteric fistula with a 2-cm abscessual cavity (external orifice h 4–5). Perianal Disease Activity Index (PDAI) was 14. EUA with partial fistulectomy and local infiltration of allogeneic mesenchymal cells (darvadstrocel) was performed while continuing the treatment with vedolizumab. At the 1-month visit, no perianal pain or secretions were reported. At 4 months, he started to experience some perianal serous secretions without pain. At that time, a colonoscopy was repeated, showing edema and ulcerations of the distal rectum and anal canal. At 6 months, pelvic MRI and TP-US confirmed the persistence of all the fistula tracts (Figures 2C,D).

### Case 2

**Figure 3:**
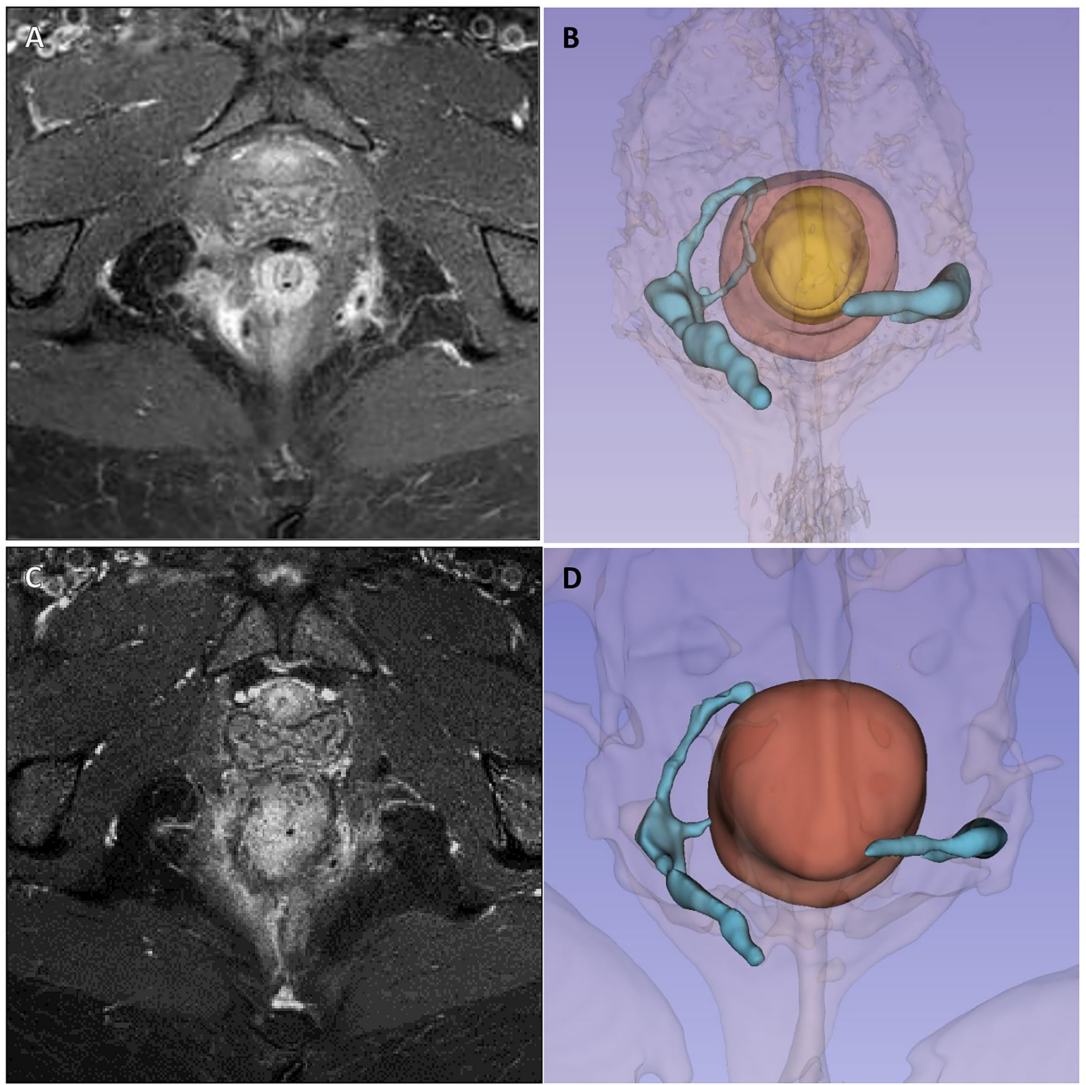
Case 2 imaging pre- and post-surgery. Panels **(A, B)** show pre-operative pelvic magnetic resonance imaging and 3D reconstruction showing two active complex trans-sphincteric fistulas: on the left a trans-sphincteric fistula with a seton in place, and on the right a branched trans-sphincteric fistula with an abscessual cavity of 17 × 10 mm in the ischioanal space and a single external orifice in the posterior right perianal quadrant (h8). Panels **(C, D)** show 6-month MRI follow-up, with persistence of fistula tracts but with less thickness and no related collections.

The second case concerns a 38-year-old woman with ileocolic and perianal Crohn's disease (A2L3B1p), and with a previous history of nervous anorexia, depressive disorder, fibromyalgia, and chronic migraine who underwent all available medical treatments for IBD starting from steroids to mesalazine, azathioprine, infliximab, adalimumab, and ustekinumab. Finally because of the persistent perianal fistulizing disease, a loop ileostomy was performed. Nevertheless, the patient continued to experience significant secretions from external perianal orifices, and a pelvic MRI performed in October 2020 showed two active complex trans-sphincteric fistulas: on the left a trans-sphincteric fistula with a seton in place, and on the right a branched trans-sphincteric fistula with an abscessual cavity of 17 × 10 mm in the ischioanal space and a single external orifice in the posterior right perianal quadrant (h8) (Figures 3A,B). An intestinal ultrasound displayed slightly increased wall thickness of the left colon with mild vascular signs at color-Doppler compatible with mildly active intestinal disease. The Perianal Disease Activity Index (PDAI) assessed was 12.

Therefore, in February 2021, she underwent EUA with perianal disease treatment and local infiltration with darvadstrocel. At 1 and 3-month visits, no secretions or pain was reported, but TP-US still detected both fistulous tracts with a linear course but without fluid collections. At 4 months, she had fistula recurrence, but the perianal abscess was successfully treated with antibiotics. Ustekinumab treatment was started again and until June she was paucisymptomatic. In August 2021, at 6 months, because of the persistence of intestinal symptoms, she underwent a subtotal colectomy with terminal ileostomy. In October 2021, 8 months after stem cell injection, she had no symptoms of perianal disease despite the radiological persistence of the tract of the fistula highlighted by an MRI scan (Figures 3C,D).

### Case 3

**Figure 4:**
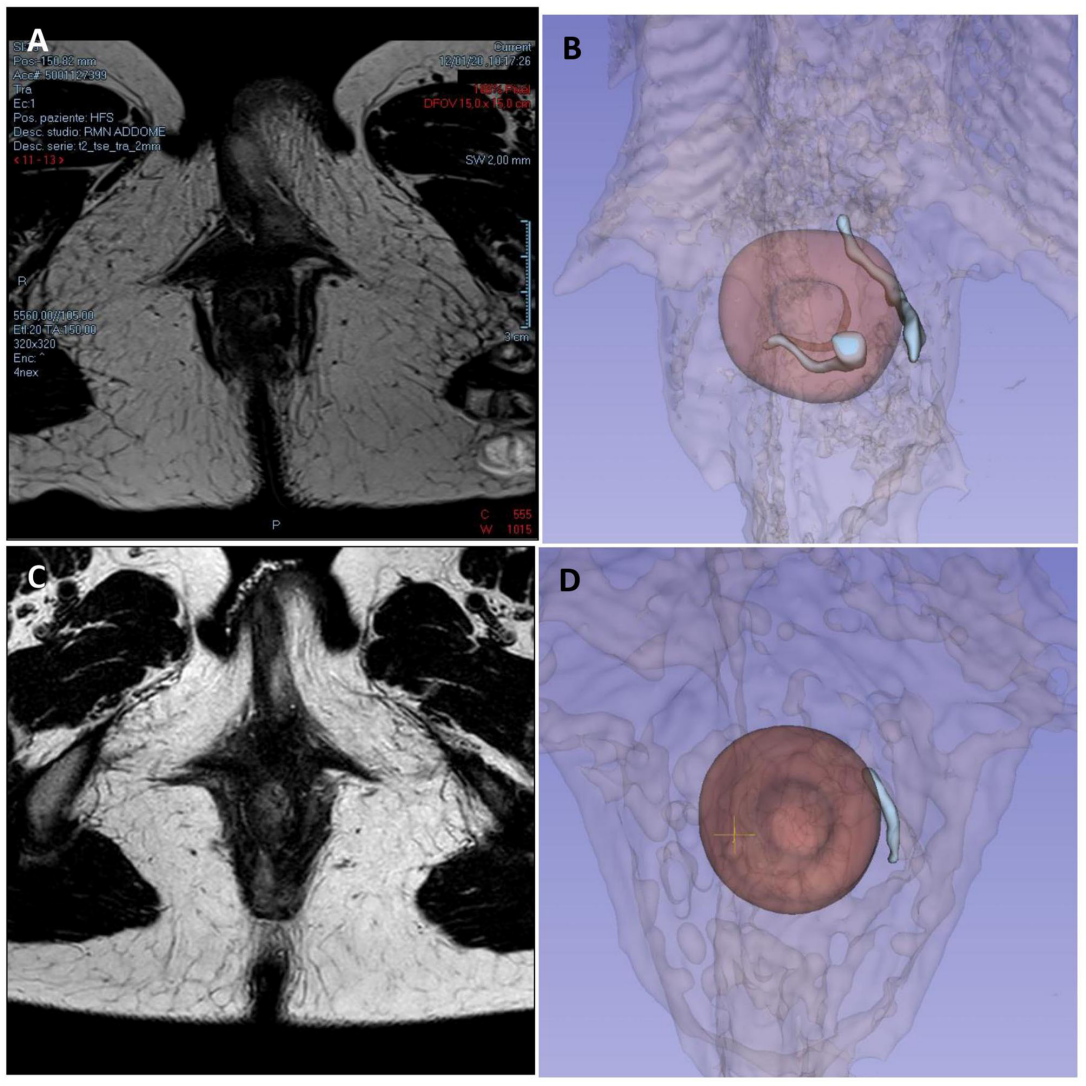
Case 3 imaging pre- and post-surgery. Panels **(A, B)** show preoperative pelvic magnetic resonance imaging and 3D reconstruction showing a trans-sphincteric fistula starting from the left wall of the anal canal going forward up to the homolateral part of the vulva, with diffuse subcutaneous tissue imbibition but without abscesses, and a fistula in the posterior wall of the anal canal with an inter-sphincteric course. Panels **(C, D)** show 6-month MRI follow-up, with the persistence of the trans-sphincteric fistula toward the vulva but without signs of inflammation or collections.

The third case is about a 28-year-old woman with ileocolic and perianal Crohn's Disease (A2L3B2p) previously treated with a double ileocolic and sigma resection with loop ileostomy and subsequent ileostomy closure with perianal abscess drainages. Medical therapy was introduced with adalimumab performed every alternating week. In October 2020, a pelvic MRI showed residual inflammation in the left paramedian perianal region, with edematous aspects and mild contrastenhancement but without fluid collections, and a trans-sphincteric deep fistula that did not reach skin layers. After 2 months, she started complaining of perianal pain and secretions, and another MRI was performed, which showed a complex fistula starting from the left wall of the anal canal at the level of the internal anal sphincter going forward up to the homolateral part of the vulva, with diffuse subcutaneous tissue imbibition but without abscesses (Figures 4A,B).

Two small secondary inter-sphincteric fistulas were also reported, one in the posterior wall of the anus with an inter-sphincteric course, and the other distally interrupting the internal sphincter and probably communicating with the previous fistula tract. The PDAI assessed was 13.

In February 2021, she underwent EUA with perianal disease conditioning and local infiltration with darvadstrocel.

At 1, 3, and 6-month visits, the perianal CD was in clinical remission, without secretions or pain, and the patient was overall in good clinical condition. TP-US at 6 months from surgery showed the persistence of the posterolateral external orifice and residual trans-sphincteric left lateral fistula. Endoscopy highlighted mild rectal reactivation of Crohn's disease. Also, the MRI performed at 6 months still displayed the fistula tract, without fluid collection and inflammatory signs (Figures 4C,D).

Table 1 summarizes the characteristics and outcomes of perianal fistulizing disease treatment of the 3 patients.

**Table 1:** Characteristics and outcomes of perianal fistulizing disease treatment.

	**Case 1**	**Case 2**	**Case 3**
**Patient**	26-year-old male	38-year-old female	28-year-old female
**Crohn's disease**	A2L2B1p	A2L3B1p	A2L3B2p
**Fistula 1 (main track)**	Right trans-sphincteric with 2 external orifices	Right branched trans-sphincteric fistula with small abscess	Left trans-sphincteric fistula with a little inter-sphincteric branch and external orifice in the labia major
**Fistula 2**	Left branched trans-sphincteric with small abscess	Left trans-sphincteric fistula with seton	/
**Previous surgery**	Abscess drainages, fistulectomies, setons	Abscess drainages, setons, loop ileostomy	Abscess drainages, ileocolic and sigma resection
**Previous medical therapy**	Adalimumab, vedolizumab,	Steroids, mesalazine, azathioprine, infliximab, adalimumab, ustekinumab	Infliximab, adalimumab
**PDAI**	14	12	13
**Symptoms**	Pain, secretions	Pain, secretions	Pain, secretions, vulvar swelling
**1 month**	No pain, no secretions	No pain, no secretions	No pain, no secretions
**3 months**	Secretions, no pain	Perianal abscess	No pain, no secretions
**>6 months**	Secretions, no pain	Subtotal colectomy with ileostomy. No perianal pain, no secretions.	No pain, no secretions
**Follow-up MRI/TP-US**	Persistence of all fistula tracts, no collections	Persistence of all fistula tracts, no collections	Persistence of fistula tracts, no collections, no inflammation
**Follow-up endoscopy**	Oedema + ulcerations distal rectum and anal canal	Pancolitis with deep ulcer sigma/rectum (before surgery)	Mild proctitis

## Discussion

The perianal fistulizing disease is one of the most common and impairing conditions affecting patients with Crohn's disease during their lifetime; thus, appropriate knowledge of specific pharmacological and surgical therapies is required (7, 8); and treatment options depend on number, localization, and complexity of fistula tracts and patient symptoms. The perianal disease severely impacts on patient's quality of life and causes substantial morbidity. Even more, approximately 70–80% of perianal Crohn's disease are complex fistulas particularly refractory to conventional medical treatment.

Despite both medical and surgical treatment options still remaining difficult and challenging, adipose-derived mesenchymal stem cells (Cx601) have proven their anti-inflammatory and immuno-modulatory potential (9–11), leading the way to the chance of being used for the treatment of fistulizing perianal CD. Stem cell therapy for perianal CD has been approved by the European Medicine Agency in 2018, but only a few studies have been conducted on the efficacy of this therapy, because of both the small number of patients and the short time of follow-up.

From a regulatory point of view, the aforementioned therapies did not receive the authorization to be supported by public institutions in Italy and other western countries, so they did not reach a wide diffusion and the clinical experience is still limited.

According to the current literature, the results of treatment with Cx601 are encouraging: half of patients with perianal CD treated with stem cell injection had clinical remission at 24 weeks, with a maximum of 56.3% at 52 weeks, as highlighted by the ADMIRE-CD study (10).

Some authors propose to perform EUA a week before stem cell injection to “clean” properly the fistula. For reasons of organization, and because we were in a “pandemic period setting”, we performed at the same time fistula conditioning and stem cell injection.

The results of our study, conducted on 3 patients treated with darvadstrocel injections for fistulizing perianal CD, have shown clinical remission (or improvement) of perianal symptoms in all the patients despite the radiological persistence of the fistulas, with no purulent secretions or abscesses after 6 months of follow-up. Although all the patients had persistent fistula tracts, perianal disease appeared to be less aggressive and debilitating, with absence of active perianal abscesses at MRI and colonoscopy, and no pain was referred by the patients.

A limitation of our report is due to the heterogeneity of our patients who presented with different clinical CD manifestations and history, sharing only a long history of refractory perianal CD, with failure to all previous medical or surgical attempts. The result of the procedure could also be influenced by the persistence of aggressive systemic CD despite strictly follow-up and use of a wide range of medications. Nevertheless, our report can fully be considered a real-life experience describing a frequent condition that clinicians have to face in a tertiary IBD care center.

Considering clinical remission, our results are in accordance with a previously reported series, where after a mean follow-up of 12 months the rate of fistula healing is around 60–70% (12).

Even if our experience is based on a small number of patients and the follow-up time is very short, the results appear encouraging and in accordance with the current literature. They demonstrate the feasibility of the procedure, its good short-term tolerance profile, and its potential efficacy.

Darvadstrocel injection seems to have a role at least in clinical remission of perianal CD, confirming its possible use in the treatment of these patients. Long-term results are still under analysis, so a complete workup still cannot be done. MRI is nowadays considered the best technique to evaluate the presence or persistence of a perianal fistula (SE: 96%, SP: 80%) (13, 14), so, since it does not expose patients to radiation, it can be considered a good diagnostic tool, giving specialists the chance to do a short, non-invasive follow-up (every 2–3 months). While MRI imaging is more objective, reproducible, and more accepted in the literature, TP-US is a useful tool that can help in obtaining a strict follow-up, and it can give complementary information to MRI. MRI 3D reconstruction can add further accurate details improving and addressing surgical fistula management during the operation.

## Conclusion

Our experience confirms the therapeutic potential of this new cellular treatment for patients with Crohn disease and refractory perianal disease. However, the mechanism promoting fistula healing is yet to be fully explored and understood.

Careful selection of patients appears to be mandatory to optimize the clinical results and obtain persistent healing of the fistula tracts. Further studies are needed to determine the real role and impact of mesenchymal stem cell administration in the treatment of complex perianal CD.

## Data Availability Statement

The raw data supporting the conclusions of this article will be made available by the authors, without undue reservation.

## Ethics Statement

Ethical review and approval was not required for the study on human participants in accordance with the local legislation and institutional requirements. The patients/participants provided their written informed consent to participate in this study. Written informed consent was obtained from the individual(s) for the publication of any potentially identifiable images or data included in this article.

## Author Contributions

FCo and FCa contributed to the design and writing of the manuscript. GM was a major contributor to data analysis and writing of the manuscript. FR participate to data collection and images production. All authors have read and approved the final manuscript.

## Conflict of Interest

The authors declare that the research was conducted in the absence of any commercial or financial relationships that could be construed as a potential conflict of interest.

## Publisher's Note

All claims expressed in this article are solely those of the authors and do not necessarily represent those of their affiliated organizations, or those of the publisher, the editors and the reviewers. Any product that may be evaluated in this article, or claim that may be made by its manufacturer, is not guaranteed or endorsed by the publisher.
